# *Campylobacter fetus* bacteremia and meningitis in an acute lymphoblastic leukemia patient undergoing maintenance therapy: a case report

**DOI:** 10.1186/s12879-021-06364-5

**Published:** 2021-07-13

**Authors:** Ryo Nakatani, Koki Shimizu, Takahiro Matsuo, Ryosuke Koyamada, Nobuyoshi Mori, Takuya Yamashita, Shinichiro Mori

**Affiliations:** 1 Department of Internal Medicine, St. Luke’s International Hospital, 9-1, Akashi-cho, Chuo-ku, Tokyo, Japan; 2 Department of Hematology, St. Luke’s International Hospital, Tokyo, Japan; 3 Department of Infectious Diseases, St. Luke’s International Hospital, Tokyo, Japan

**Keywords:** *Campylobacter fetus*, Ceftriaxone, Meropenem

## Abstract

**Background:**

*Campylobacter fetus* is an uncommon *Campylobacter* species*,* and its infections mainly cause infective endocarditis, aortic aneurysm, and meningitis rather than enteritis. It is more likely to be detected in blood than *Campylobacter jejuni* or *Campylobacter coli*, specifically reported in 53% of patients. In our case, *C. fetus* was detected in both blood and cerebrospinal fluid (CSF) cultures.

**Case presentation:**

A 33-year-old woman, who was on maintenance chemotherapy for acute lymphoblastic leukemia (ALL), presented to our clinic with chief complaints of severe headache and nausea. Blood and CSF cultures revealed *C. fetus*. We administrated meropenem 2 g intravenously (IV) every 8 h for 3 weeks, and she was discharged without neurological sequelae.

**Conclusion:**

We encountered a case of *C. fetus* meningitis without gastrointestinal symptoms, neck stiffness or jolt accentuation in a patient with ALL. Undercooked beef was considered the source of *C. fetus* infection in this case, suggesting that the need for a neutropenic diet and safe food handling be considered.

## Background

*Campylobacter fetus* is an uncommon *Campylobacter* species and its infections are mainly infective endocarditis [1], aortic aneurysm [2], and meningitis [3] rather than enteritis. It is likely to be detected in blood than *Campylobacter jejuni* or *Campylobacter coli*, specifically reported in 53% of patients [4]. In our case, *C. fetus* was detected in both blood and cerebrospinal fluid (CSF) cultures.

## Case presentation

A 33-year-old woman, who was on maintenance chemotherapy for acute lymphoblastic leukemia (ALL), presented to our clinic with chief complaints of severe headache and nausea. She had been diagnosed with ALL 2 years before this event and was in the process of receiving maintenance treatment under the Japan Adult Leukemia Study Group ALL202-U protocol [5]. Her headache and nausea had started after receiving an intrathecal chemotherapy (methotrexate, cytarabine, and prednisolone) 2 weeks before; thus, she initially interpreted the headache as post-lumbar-puncture pain and consumed painkillers (loxoprofen and tramadol) that partially relieved her symptoms. However, her symptoms continued, and by the time of her visit to our clinic, she also developed other symptoms, such as photosensitivity, appetite loss, left temporal and eye pain, and tinnitus. She had no gastrointestinal symptoms or fever during the 2 weeks. She had no medical history before her diagnosis of ALL and had only one episode of septic shock with lung abscess during her course of chemotherapy, which was caused by *Klebsiella pneumoniae*. She was on prophylactic antimicrobials, including atovaquone (leukopenia due to trimethoprim-sulfamethoxazole), fluconazole, and acyclovir. Notably, she had eaten undercooked beef 2 days prior to admission. On admission, the patient experienced acute distress, had a body temperature of 36.2 °C, blood pressure of 130/88 mmHg, pulse rate of 87/min, respiratory rate of 18/min, and oxygen saturation measured by pulse oximetry of 100% on room air. The patient did not have neck stiffness, jolt accentuation, or any other neurologic deficits, such as cranial nerve abnormality, ataxia, or muscle weakness in her extremities. She had no abdominal tenderness or normoactive bowel sounds. Laboratory findings were the following: white blood cell count, 2400/μL (neutrophils, 58%; lymphocytes, 27.5%; monocytes, 11.5%); hemoglobin level, 11.5 g/dL; platelet count, 336,000/μL; blood urea nitrogen level, 10.2 mg/dL; creatinine level, 0.48 mg/dL; and C-reactive protein level, 3.74 mg/dL. Computed tomography of the brain did not reveal any abnormalities. Incidentally, her neutrophil count had been hovering around 1000/μL, a month prior to her admission. Her headache was so intense that we started intravenous patient-controlled analgesia with fentanyl.

**Fig. 1:**
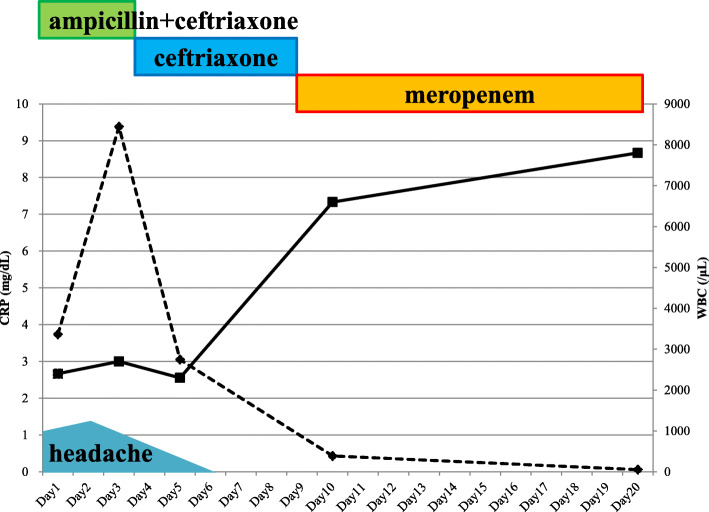
WBC level (solid line) and CRP level (dotted line) after patient admission. WBC: white blood cell, CRP: C-reactive protein

On day 2, her temperature rose to 38.0 °C and jolt accentuation was observed. Lumbar puncture was performed, and CSF analysis showed a cell count of 71 (polymorphonucleocytes 59.2% and mononucleocytes 40.8%), protein level of 87 mg/dL, and glucose level of 82 mg/dL (serum glucose 124 mg/dL). No bacteria were observed under gram staining. We empirically initiated ampicillin 2 g intravenously (IV) every 4 h and ceftriaxone IV 2 g every 12 h. Her symptoms gradually improved. On day 4, spiral gram-negative rods were detected in blood cultures obtained on admission, and ampicillin was discontinued. On day 6, blood and CSF cultures revealed *C. fetus*, which was identified by an aerobic bottle, BacT/ALERT (bioMérieux, Inc., Durham, NC). The minimum inhibitory concentrations (MICs) of this strain measured by E-test (bioMérieux) were as follows: meropenem, 0.064 μg/mL and cefotaxime, 16 μg/mL. Stool cultures were negative on day 9, and we switched from ceftriaxone to meropenem 2 g IV every 8 h (Fig. 1). We excluded other metastatic foci, such as infective endocarditis, splenic or renal abscess, septic pulmonary emboli, or infected aneurysm. We continued meropenem for 3 weeks, and she was discharged without neurological sequelae.

## Discussion and conclusions

We report on a case of *C. fetus* bacteremia and meningitis successfully treated with meropenem in a patient with ALL.

Notably, the patient consumed undercooked beef and subsequently developed meningitis, as previously reported [6]. Although she denied any abdominal pain or diarrhea, considering that approximately half of the patients with *C. fetus* meningitis were without any gastrointestinal symptom, we assumed that the origin of infection could be the gastrointestinal tract [3, 7]. *C. fetus* is a part of the normal flora in the gastrointestinal tracts of sheep and cattle [8]. A previous study reported 38% of the 19 cases had frequent contact with domestic animals [3] and 62–73% cases of *C. fetus* bacteremia or meningitis had a significant underlying disease, such as diabetes, alcoholism, or cardiovascular diseases [3, 9]. Since this patient underwent maintenance therapy for ALL, the white blood cell count on admission was low, especially a neutrophil count of approximately 1000/μL was noted for at least 1 month, and the patient was considered immunocompromised. On the contrary, immunocompetent individuals also experience *C. fetus* bacteremia [10, 11], and we should always pay attention when spiral gram-negative rods are obtained on blood culture.

In previous studies, a neutropenic diet has referred to a low bacterial diet, and neutropenic patients have been instructed to avoid mainly fresh fruits or vegetables [12, 13]. A recent study showed that a neutropenic diet was not associated with a reduced risk of infection in neutropenic patients [14]; therefore, we did not instruct the patient to adhere to a neutropenic diet. However, safe food handling [15] may have been insufficient, and the entry of *C. fetus* was due to lack of instruction to the patient. In Japan, eating raw or undercooked meat is customary and the guidance on safe food handling is seemingly not thorough. Since food habits in Japan are often different from those in Western countries, it was considered necessary to create Japanese versions of safe food handling protocols. Furthermore, owing to limited evidence regarding consumption of undercooked meat or fish among neutropenic patients, further studies are needed to establish safe management of immunocompromised patients.

Since the patient did not have fever or neck stiffness or jolt accentuation, lumbar puncture was not performed on the day of admission. We considered that central nervous system infiltration of ALL or chemical meningitis was the primary differential diagnosis. However, she developed fever and was noted to present these signs on the second day; thus, we performed lumbar puncture and confirmed the diagnosis of meningitis. According to previous studies, clinical manifestations of *C. fetus* meningitis vary and only 64 and 59% had headache and neck stiffness, respectively, although fever was noted in approximately 90% of patients [3, 8]. Therefore, even if the patient has no neck stiffness or neurological abnormalities, clinicians should consider the possibility of *C. fetus* meningitis in immunocompromised or immunocompetent patients with high-risk dietary habits.

The recommended antimicrobials and duration of treatment for *C. fetus* infections are yet to be established. Previous studies reported the susceptibilities of *C. fetus* (MIC_90_) were as followed: meropenem 0.12 μg/mL, imipenem ≤0.06 μg/mL, cefotaxime 16–64 μg/mL, ampicillin 2–32 μg/mL, gentamycin 1–2 μg/mL, tetracycline 0.5–128 μg/mL, and ciprofloxacin 0.5–1 μg/mL [16–18]. The lowest MICs were obtained with imipenem and meropenem. Cefotaxime is not an appropriate antimicrobial for *C. fetus* infection, since lower bactericidal activities in vitro were reported than those of ampicillin, gentamicin, and imipenem [14]. In accordance with this and E-test results, we switched from ceftriaxone to meropenem and continued the treatment for 3 weeks. Meropenem 2 g IV every 8 h would be the first choice for *C. fetus* meningitis, considering imipenem poses a risk of causing seizure [19]. Although intrathecal therapy for meningitis is sometimes considered [20], there are no reports for *C. fetus* meningitis. Since it would be a possible treatment option, more cases need to be accumulated.

In conclusion, we encountered a case of *C. fetus* meningitis without gastrointestinal symptoms, neck stiffness, or jolt accentuation in a patient with ALL. Undercooked beef was considered the source of *C. fetus* infection in this case, suggesting that the need for a neutropenic diet and safe food handling be considered. No standard treatment for *C. fetus* infection has been established, and its susceptibility to antibacterial agents is insufficient. Further studies combining clinical symptoms and susceptibility data are warranted.

## Data Availability

Not applicable.

## Abbreviations

ALL: Acute lymphoblastic leukemia
CSF: Cerebrospinal fluid
IV: Intravenously
MICs: Minimum inhibitory concentrations

## References

[1] Caramelli BR, Mansur AJ, Grinberg M, Mendes CM, Pileggi FU (1988). Campylobacter fetus endocarditis on a prosthetic heart valve. South Med J.

[2] Marty AT, Webb TA, Stubbs KG, Penkava RR (1983). Inflammatory abdominal aortic aneurysm infected by campylobacter fetus. JAMA.

[3] van Samkar A, Brouwer MC, van der Ende A, van de Beek D (2016). Campylobacter fetus meningitis in adults: report of 2 cases and review of the literature. Medicine.

[4] Pacanowski J, Lalande V, Lacombe K, Boudraa C, Lesprit P, Legrand P, Trystram D, Kassis N, Arlet G, Mainardi JL, Doucet-Populaire F, Girard PM, Meynard JL, CAMPYL Study Group (2008). Campylobacter bacteremia: clinical features and factors associated with fatal outcome. Clin Infect Dis.

[5] Hayakawa F, Sakura T, Yujiri T, Kondo E, Fujimaki K, Sasaki O (2014). Markedly improved outcomes and acceptable toxicity in adolescents and young adults with acute lymphoblastic leukemia following treatment with a pediatric protocol: a phase II study by the Japan Adult Leukemia Study Group. Blood Cancer J.

[6] Suy F, Le Dû D, Roux AL, Hanachi M, Dinh A, Crémieux AC (2013). Meningitis and endocarditis caused by campylobacter fetus after raw-liver ingestion. J Clin Microbiol.

[7] Brah S, Chiche L, Brun M, Schleinitz N, Harle JR, Durand JM (2011). Campylobacter fetus bacteremia revealed by cellulitis without gastrointestinal symptoms in the context of acquired hypogammaglobulinemia: a report of three cases. Case Rep Gastrointest Med.

[8] Patrick ME, Gilbert MJ, Blaser MJ, Tauxe RV, Wagenaar JA, Fitzgerald C (2013). Human infections with new subspecies of campylobacter fetus. Emerg Infect Dis.

[9] Gazaigne L, Legrand P, Renaud B, Bourra B, Taillandier E, Brun-Buisson C, Lesprit P (2008). Campylobacter fetus bloodstream infection: risk factors and clinical features. Eur J Clin Microbiol Infect Dis.

[10] Nagy MT, Hla SM (2013). Campylobacter fetus sepsis in an immunocompetent patient with haematological complication. BMJ Case Rep.

[11] Fernández-Cruz A, Muñoz P, Mohedano R, Valerio M, Marín M, Alcalá L, Rodriguez-Créixems M, Cercenado E, Bouza E (2010). Campylobacter bacteremia: clinical characteristics, incidence, and outcome over 23 years. Medicine.

[12] Wison BJ (2002). Dietary recommendations for neutropenic patients. InSemin Oncol Nurs.

[13] Moody K, Finlay J, Mancuso C, Charlson M (2006). Feasibility and safety of a pilot randomized trial of infection rate: neutropenic diet versus standard food safety guidelines. J Pediatr Hematol Oncol.

[14] Ball S, Brown TJ, Das A, Khera R, Khanna S, Gupta A (2019). Effect of neutropenic diet on infection rates in cancer patients with neutropenia. Am J Clin Oncol.

[15] US Food and Drug Administration (2019). Food safety at home.

[16] Tremblay C, Gaudreau C (1998). Antimicrobial susceptibility testing of 59 strains of campylobacter fetus subsp. fetus. Antimicrob Agents Chemother.

[17] Tremblay C, Gaudreau C, Lorange M (2003). Epidemiology and antimicrobial susceptibilities of 111 campylobacter fetus subsp. fetus strains isolated in Quebec, Canada, from 1983 to 2000. J Clin Microbiol.

[18] Vandenberg O, Houf K, Douat N, Vlaes L, Retore P, Butzler JP, Dediste A (2006). Antimicrobial susceptibility of clinical isolates of non-jejuni/coli campylobacters and arcobacters from Belgium. J Antimicrob Chemother.

[19] Kim BN, Peleg AY, Lodise TP, Lipman J, Li J, Nation R, Paterson DL (2009). Management of meningitis due to antibiotic-resistant Acinetobacter species. Lancet Infect Dis.

[20] Tunkel AR, Hasbun R, Bhimraj A, Byers K, Kaplan SL, Scheld WM, van de Beek D, Bleck TP, Garton HJL, Zunt JR (2017). Infectious Diseases Society of America's clinical practice guidelines for healthcare-associated ventriculitis and meningitis. Clin Infect Dis.

